# To Study the Correlation of Clinical Severity and Cytokine Storm in COVID-19 Pulmonary Embolism Patients by Using Computed Tomography Pulmonary Angiography (CTPA) Qanadli Clot Burden Scoring System

**DOI:** 10.7759/cureus.39263

**Published:** 2023-05-20

**Authors:** Liaquat Ali, Muhammad Sharif, Syed Ghafran Ali Naqvi, Imran Mohammed, Mirza A Baig, Kazi Sidratul Muntaha, Ameena R Chalil, Hanna Ali, Hana a Aweida, Ambreen Iqrar

**Affiliations:** 1 Neurology, Hamad General Hospital, Doha, QAT; 2 Neurology, Weill Cornell Medicine-Qatar, Doha, QAT; 3 Internal Medicine, Hamad General Hospital, Doha, QAT; 4 Radiology, Hamad General Hospital, Doha, QAT; 5 Medicine, Hamad General Hospital, Doha, QAT; 6 Neurology, Aga Khan University Hospital, Karachi, PAK

**Keywords:** ct pulmonary angiogram (ctpa), severe acute respiratory syndrome coronavirus-2 (sars-cov-2), coronavirus disease -2019 (covid-19), covid-19-associated coagulopathy (cac), neutrophil extracellular traps (nets), disseminated intravascular coagulation (dic), deep venous thrombosis (dvt), venous thromboembolism (vte), pulmonary embolism (pe)

## Abstract

Background: Pulmonary embolism (PE) is a fatal form of venous thromboembolism (VTE), with an overall untreated mortality of up to 30%. Greater than 50% of patients with lower extremity proximal DVT have concurrent PE at presentation. VTE has been seen in up to one-third of patients with COVID-19 infections requiring intensive care unit (ICU) admission. The objective of this study is to determine the correlation between CT pulmonary angiography, pulmonary embolism clot burden, and the Qanadli scoring system with clinically severe COVID-19 pneumonia and cytokine storm.

Material and Method: 153 COVID-19 hospitalized patients who underwent CT pulmonary angiography (CTPA) for likely PE on pretest probability modified Wells criteria were enrolled. COVID-19 pneumonia was classified as URTI (upper respiratory tract infection), mild, severe, and critical COVID pneumonia. For data analysis, we categorized into two groups: (1) the non-severe group included URTI and mild pneumonia, and (2) the severe group included severe and critical pneumonia. We used the Qanadli scoring system to assess the PE percentages of pulmonary vascular obstruction using CTPA.

Results: 41.8% (64) of COVID-19 patients were diagnosed with pulmonary embolism (PE) on CTPA. The majority of 51.6% of pulmonary vascular occlusions using the Qanadli scoring system for pulmonary embolism were at segmental arterial levels. Out of 104 COVID-19 cytokine storm patients, 45 (43%) were associated with pulmonary embolism. Overall, a 25% (16) mortality rate was observed in COVID-19 patients with pulmonary embolism.

Discussion: The pathogenesis of hypercoagulability in COVID-19 may include direct endothelial cell invasion by the virus, microvascular inflammation, endothelial exocytosis, and endotheliitis. A meta-analysis of 71 studies to investigate the occurrence of PE on CTPA in COVID-19 patients found 48.6% in ICU settings and 65.3% of patients have clots in the peripheral pulmonary vasculature.

Conclusions: There is a significant correlation between pulmonary embolism and high clot burden Qanadli CTPA scores, as well as between the severity of COVID-19 pneumonia and mortality. The association between critically ill COVID-19 pneumonia and pulmonary embolism may result in higher mortality and a poor prognostic marker.

## Introduction

Pulmonary embolism (PE) may be an occasionally fatal form of venous thromboembolism (VTE). The overall incidence of PE is approximately 112 cases per 100,000, increases with age, and is somewhat more common in males than females [1,2]. PE refers to partial or complete obstruction of the pulmonary artery or one of its branches by material such as a thrombus, tumor, air, or fat that originated somewhere else in the body. PE may be classified as acute, subacute, chronic, hemodynamically unstable, or stable, and by anatomic location such as saddle, lobar, segmental, and sub-segmental [3-5]. 

The pathogenesis of PE included endothelial injury, a hypercoagulable state, and venous stasis (Virchow’s triad). Most PE arises from the proximal veins of the lower extremities (popliteal, femoral, and iliac), and more than 50% of proximal deep vein thrombosis (DVT) patients have coexisting PE at the time of presentation [6-10]. In most cases, PE is typically multiple, with the lower lobes being involved [11]. A series of pathophysiologic changes may occur once a thrombus lodge in the lung, including pulmonary infraction, impaired gas exchanges, and cardiovascular compromise with right heart failure [12-14]. The most common symptoms of PE are dyspnea, followed by pleuritic chest pain, coughing, and the sign of DVT. The life-threatening PE may require thrombolysis, inferior vena cava filters, and embolectomy in addition to anticoagulation.

VTE is common in up to one-third of critically ill COVID-19 patients, even on prophylactic anticoagulation [15]. The disseminated intravascular coagulation (DIC)-like the state is referred to as a COVID-19-associated hypercoagulable state [16, 17]. VTE prophylaxis is the standard of care in all COVID-19 hospitalized patients and intensive care units (ICU) unless there is a contraindication to anticoagulation. The primary objective of this study is to determine the correlation between the percentage of PE clot burden by using the Qanadli scoring system for pulmonary vascular obstruction in CTPA and the clinical severity of COVID-19 pneumonia, and the secondary objective is to assess the correlation between the percentage of PE clot burden in CTPA and cytokine storm.

## Materials and methods

This is an observational, cross-sectional, retrospective cohort study of charts/data reviews of COVID-19 patients admitted under Hamad Medical Cooperation (HMC) in different multiple tertiary care COVID-19 hospital facilities from 01/03/2020 to 30/04/2021. The study was approved by the Ethics Committee of HMC (MRC-01-21-415), Qatar, on June 14, 2021, and written informed consent was waived due to its retrospective nature, the rapid emergence of COVID-19, and the urgent need for data collection. After approval by the HMC Ethics Committee, we retrospectively review charts/data of admitted COVID-19 patients who underwent computed tomography (CT) pulmonary angiography. A total sample size of 153 COVID-19 patients with confirmed COVID-19 by using a real-time reverse transcription-polymerase chain reaction (RT-PCR) swab and a CT pulmonary angiography with a suspected pretest probability of likely PE on modified Wells criteria were included. Patients with advanced lung neoplasms, a history of lobectomy, tuberculosis, and severe atelectasis were excluded from the study. According to the CDC Qatar’s recommendation for diagnosis and treatment of COVID-19 pneumonia, it is classified as URTI (upper respiratory tract infection), mild, severe, and critical COVID pneumonia. For data analysis, we categorized into two groups: (1) the non-severe group included URTI and mild pneumonia, and (2) the severe group included severe and critical pneumonia. Patients’ data/electronic charts were reviewed by a trained physician, and baseline investigations, including chest X-ray, inflammatory markers (Ferritin, CRP, D-dimer, interleukin-6 (IL-6)), the comprehensive metabolic panel (CMP), and CT pulmonary angiography findings, were recorded on a dedicated MS Excel (Redmond, USA) datasheet. Images were reviewed by a consultant radiologist on a radiology workstation in settings for pulmonary vasculature and lung parenchyma. The presence or absence of occlusive or non-occlusive right ventricle thrombus, main pulmonary artery trunk, right or left pulmonary artery, inter-lobar pulmonary artery, lobar, segmental, sub-segmental, and intra-lobar arteries were recorded. All CT pulmonary angiograms were read by a consultant radiologist, and PE clot burden scores were calculated for all PE-positive CT pulmonary angiograms. We used the Qanadli scoring system based on the site of obstruction and the degree of occlusion of the pulmonary arteries. According to the Qanadli scoring system, the pulmonary arterial tree of each lung is regarded as having 10 segmental pulmonary arteries (three to the upper lobes, two to the middle lobe or lingula, and five to the lower lobes). The presence of an embolus in a segmental pulmonary artery is scored as 1 point, and emboli at the most proximal arterial level have a score equal to the number of segmental pulmonary arteries arising distally. To provide additional information on the residual perfusion distal to an embolus, a weighting factor is used for each value (0=no defect, 1=partial occlusion, 2=complete occlusion). An isolated sub-segmental embolus is considered a partially occluded segmental pulmonary artery and is assigned a value of 1. The maximum CT PE clot obstruction index is 40. The CT pulmonary angiogram severity score is based on the percentage of the obstructed surface of pulmonary arteries and used on a four-point scale (1=0-24%, 2=25-49%, 3= 50-74%, 4=75-100%). The data were collected from the electronic patient record system (Cerner) on a pre-designed MS Excel (Redmond, USA) data collection sheet by a dedicated team.

This study aims to determine the mortality rate in COVID-19 patients with PE clot burden severity by using the Qanadli scoring system. Descriptive statistics are used to summarize and determine the sample characteristics and distribution of various considered parameters related to demographic, diagnostic, clinical, follow-up outcome measures, and other related features of such patients. The normally distributed data and results were reported with a mean and standard deviation (SD) with a corresponding 95% confidence interval (CI); the remaining results were reported with a median and interquartile range (IQR). Categorical data are summarized using frequencies and percentages. Associations between two or more qualitative variables were examined and assessed using Pearson Chi-square and Fisher Exact tests, as appropriate. Unpaired-T test and ANOVA were used to compare the mean values of different quantitative parameters between two or more groups. The correlation between various outcomes measured and evaluated quantitatively is calculated by Pearson or Spearman rank-order correlation. Appropriate regression analysis was performed to assess the effects of various factors and co-variates on primary outcome measures. Pictorial presentations of the key results were made using appropriate statistical graphs. A two-sided p-value of <0.05 is statistically significant. All statistical analyses were done using IBM Corp. Released 2016. IBM SPSS Statistics for Windows, Version 24.0. Armonk, NY: IBM Corp.

## Results

A total of 153 COVID-19 hospitalized patients who were suspected of PE and had a CT pulmonary angiogram performed were enrolled in this study. Demographic and clinical characteristics are shown in Table 1.

**Table 1:** Demographics and clinical characteristics Demographics and clinical characteristics, including age, gender, COVID-19 PCR, symptoms, risk factors, severity of CAP, and Wells criteria for PE.

Variables	Frequency(n=153)	Percentage (%)
Age (Mean=54 years)	153 patients (range-28-84)	
Male	141	92%
Female	12	8%
COVID-19 RT-PCR test Results		
COVID-19 RT-PCR test positive	141	92%
COVID-19 RT-PCR test reactive	12	8%
Symptoms		
Fever	124	81%
Cough	113	73.9%
SOB	112	73.2%
Myalgia	31	20.3%
Risk Factor		
Diabetics (DM-2)	71	46.7%
Hypertension (HTN)	70	46.1%
Obesity (BMI>30)	34	22%
Newly diagnosed DM-2	19	12.5%
Community-Acquired Pneumonia (ATS)		
Mild	8	5.3%
severe	18	11.7%
critical	127	83%
Wells’ Criteria for PE pre-test probability		
Likely PE	131	85.6%
Unlikely PE	22	14.37%
Odds ratio for PE likely and unlikely wells criteria for pretest probability.	2.79 (OR)	

The mean age was 54 years (ranging from 28 to 84 years), 92% (141) were males, and 8% (12) were females. 92% (141) had positive COVID-19 RT-PCR swab tests, while 8% (12) were reactive. The most common symptoms at the onset of illness included fever 81% (124), cough 73.9% (113), shortness of breath 73.2% (112), myalgia 20.3% (31), headache 5.9% (9), chest pain 3.9% (6), and anosmia 2% (3) (Table 1). Underlying risk factors included 46.7% (71) being diabetic, 46.1% (70) being hypertensive, 22% (34) being obese with >30 BMI, 12.5% (19) being newly diagnosed with diabetes, 7.9% (12) being pre-diabetic, 6.6% (10) being chronic kidney disease (CKD), 6.6% (10) being ischemic heart disease (IHD), 3.9% (six) being asthmatic, and 2.6% (four) being smokers (Table 1). According to the American Thoracic Society guidelines for community-acquired pneumonia (CAP), 83% of patients (127) were critically ill, 11.7% (18) had severe pneumonia, and 5.3% (eight) had mild pneumonia. Wells’ Criteria for risk stratifies for PE pre-test probability of likely was 85.6% (131) and unlikely was 14.37% (22) with an odd ratio (OR) of 2.79. CT pulmonary angiography showed that 41.8% (64) patients were positive for pulmonary embolism, and 23.53% (36) had bilateral pulmonary embolism (Table 2). In the CT pulmonary angiogram, 51.6% (33) were vascular occlusions at the pulmonary segmental arteries levels, 10.9% (seven) were sub-segmental arteries level, 10.9% (seven) were pulmonary artery levels, 6.3% (four) were lobar arteries levels, 3.1% (two) were inter-lobar arteries levels, and 1.5% (one) were clots at the main pulmonary trunk level, as in descending order (Table 2). According to the Qanadli scoring system for PE obstruction and degree of occlusion of pulmonary arteries, the majority of pulmonary embolisms involved lower lobe segmental pulmonary arteries. In this study, the CT pulmonary angiogram obstruction severity score is based on the percentage (%) of obstruction of the pulmonary arteries. In this study, the percentage of pulmonary obstruction severity scores from 0% to 24% was 78.1% (50), from 25% to 49% was 10.9% (seven), from 50% to 74% was 6.3% (four), and from 75% to 100% was 4.68% (three). The majority of Qanadli scoring systems for PE obstruction severity scores in percentages from 0% to 24% were 78.1% (50) (Table 2).

**Table 2:** CT pulmonary angiography Qanadli scoring system for obstruction and the degree of occlusion of the pulmonary arteries. CT pulmonary angiography for pulmonary embolism at different pulmonary arterial systems and Qanadli scores for the degree of occlusion of the pulmonary arteries in percentages (%).

CT pulmonary angiography for pulmonary embolism	Frequency (n)	Percentage (%)
Diagnosed pulmonary embolism (PE)	64	41.8%
PE segmental arteries level	33	51.6%
Bilateral pulmonary embolism (PE)	36	23.53%
Qanadli score for the degree of occlusion of the pulmonary arteries in % (n=64)		
Degree of occlusion of the pulmonary arteries from 0 to 24%	50	78.1%
Degree of occlusion of the pulmonary arteries from 25% to 49%	7	10.9%
Degree of occlusion of the pulmonary arteries from 50% to 74%	4	6.3%
Degree of occlusion of the pulmonary arteries from 75% to 100%	3	4.68%

**Figure 1:**
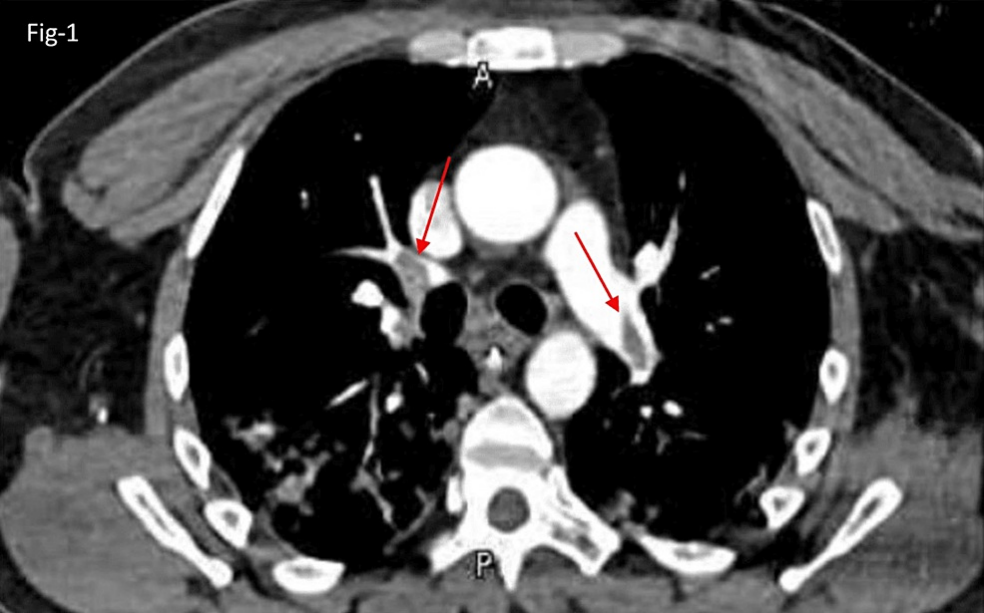
CTPA showed PE in bilateral main pulmonary arteries and extended lobar branches. Bilateral pulmonary embolism in the main pulmonary arteries extends into all lobar arteries and their multiple segmental branches.

Figure 1 of CTPA showed pulmonary embolism in bilateral main pulmonary arteries extending into all lobar and their multiple segmental branches (as shown with a red arrowhead) and bilateral patchy ground glass opacities.

Various laboratory parameters associated with the severity of COVID-19 infection included 95.4% (145) high LDH, 95.4% (145) high ferritin level, 90.8% (138) high C-reactive protein, 88.2% (134) high D-dimer, and 68.4% (104) high IL-6 (Table 3). 

**Table 3:** Laboratory features for severe COVID-19 and other multiorgan system involvement. Various laboratory parameters such as LDH, ferritin, CRP, D-dimer, and IL-6 associated with the severity of COVID-19 infection and other multiorgan system involvement were included.

Lab: Value	Frequency (n=153patients)	Percentage (%)
LDH (>245 units/L associated with severe COIVD-19)	145	95.4%
Ferritin level (>500 mcg/L associated with severe COIVD-19)	145	95.4%
C-reactive protein (>100 mg/l associated with severe COIVD-19)	138	90.8%
D-dimer (>3000 ng/mL associated with severe COIVD-19)	134	88.2%
Serum CK (>300u/L associated with severe COIVD-19)	122	80.3%
IL-6 (>70 pg/ml associated with cytokine storm)	104	68.4%
high troponin T hs (For males>30, females>20ng/l associated with severe COIVD-19 and acute myocardial injury)	79	52%
Procalcitonin (> 2ng/ml associated with severe COIVD-19)	79	52%
Acute Kidney injury (AKI) (abnormal creatinine >106umol/l and urea >8mmol/l)	69	45.4%
ALT or AST (>200 U/L associated with severe COIVD-19 and Acute liver injury)	61	40.1%
Lactic acid (> 4mml/l associated with severe sepsis)	13	8.6%
Serum lipase (>180 u/l associated with acute pancreatitis)	10	6.6%
HbA1c (values equal and greater >12 associated poor control DM)	5	3.3%

A Pearson correlation was conducted to examine relationships between CTPA positive or negative PE, CTPA PE severity score obstructed in percentages, severe COVID-19 cytokine storm, severe COVID-19 pneumonia and hospital discharge. There is a significant Pearson correlation between CTPA-PE and CTPA-PE severity score obstructed in percentages r (153)=0.793, P<0.01 level (2-tailed), as well as between severity COVID-19 pneumonia, hospital discharge, or death r (153)=0.186, P<0.022 level (2-tailed) (Table 4). 

**Table 4:** Pearson correlation between CTPA for PE, severe COVID-19 cytokine storm, CTPA PE severity score obstructed in %, severe COVID-19 pneumonia and discharge. Pearson correlation between CTPA positive or negative PE and severe COVID-19 cytokine storm, CTPA PE severity score obstructed in % (1% to 24%, 25% to 49%, 50% to 74%, 75% to 100%), severe COVID-19 pneumonia (URTI, mild, severe, critical, ARDS, sepsis/septic shock), and discharge (still hospital, home, rehab, death).

		CTPA-PE	CTPA-PE severity score % obstructed	Cytokine Storm	Pneumonia	Discharge
CTPA-PE	Pearson Correlation	1	.793^**^	.083	.036	-.096
	Sig. (2-tailed)		< .001>	.308	.657	.242
	N	153	153	153	153	153
CTPA-PE severity score % obstructed	Pearson Correlation	.793^**^	1	.066	-.043	-.110
	Sig. (2-tailed)	< .001>		.420	.596	.178
	N	153	153	153	153	153
Cytokine Storm	Pearson Correlation	.083	.066	1	-.018	-.014
	Sig. (2-tailed)	.308	.420		.823	.867
	N	153	153	153	153	153
Pneumonia	Pearson Correlation	.036	-.043	-.018	1	.186^*^
	Sig. (2-tailed)	.657	.596	.823		.022
	N	153	153	153	153	153
Discharge	Pearson Correlation	-.096	-.110	-.014	.186^*^	1
	Sig. (2-tailed)	.242	.178	.867	.022	
	N	153	153	153	153	153
**. Correlation is significant at the 0.01 level (2-tailed)						
*. Correlation is significant at the 0.05 level (2-tailed).						

A total of 151 patients were admitted to medical intensive care units (MICU), and 124 patients (81%) required intubation and mechanical ventilation during the hospital stay. 35.9% (23) were PE-positive. COVID-19 patients were discharged home; 29.6% (19) were transferred to the rehabilitation center, and 25% (16) died. 9.4% (six) of COVID-19 patients were still in the hospital during the study period. 92% (59) of patients with PE positive had severe (severe to critical) COVID-19 pneumonia, while only 8% (five) had non-severe (URTI to mild) COVID-19 pneumonia. 43% (45) of cytokine storm patients were PE-positive. 31% (14) of PE positives associated with cytokine storms died, while 23.5% (15) of PE positives with critical or severe COVID-19 pneumonia died. There was a higher rate of mortality observed in PE-positive severe and critical COVID pneumonia and with cytokine storm (Table 5). 

**Table 5:** PE patients admitted to the MICU have a mortality rate and high inflammatory and cytokine markers. MICU-admitted patients and severely high inflammatory or cytokine markers in confirmed pulmonary embolisms.

MICU Admitted Patients	Frequency (n=151)	Percentage (%)
Patient mechanical ventilated (with or without PE cases)	124	81%
PE-diagnosed cases were mechanically ventilated	50	40.3%
PE associated with critical/ severe pneumonia	59	40.69%
PE associated with critical/severe pneumonia died	15	23.5%
PE associated with cytokine storm	45	43.3%
PE with cytokine storm died	14	31%
Pulmonary embolism with severely high inflammatory markers and cytokine markers		
High D dimer with PE (>3mg/L associated with severe COVID-19)	59	44%
High IL-6 with PE (>70pg/ml associated with severe cytokine storm)	45	43.26%
High CRP with PE (>100mg/L associated with severe COVID-19)	59	42.75%
High Ferritin with PE (>500ng/ml associated with severe COVID-19)	59	40.68%
High LDH with PE (>245U/L associated with severe COVID-19)	56	38.62%

To control cytokine storm in patients with IL-6> 70 pg/ml, 100 patients (65.4%) received standard dose steroids, and different immunomodulators were given, including 51 patients (33%) who received anakinra (IL-1 antagonist), 47 patients (30.7%) tocilizumab (IL-6 antagonist), 29 (18.95%) patients who received convalescent plasma, and 27 patients (17.6%) received IVIG.

## Discussion

This is a retrospective cohort cross-sectional observational study of 153 COVID-19 hospitalized patients whose charts/data were reviewed and who underwent CT pulmonary angiograms for suspected PE. The majority of these hospitalized COVID-19 patients were critically ill (83%), with severe pneumonia (11.7%). 41.8% (64) of patients had pulmonary embolism on CTPA, and 23.5% (36) had bilateral pulmonary involvement. The odd ratio of 2.79 (OR) for likely PE on Wells’ criteria for pretest probability risk for PE. In this study, according to the Qanadli CT pulmonary angiogram, PE obstruction severity scores from 0 to 24% were 78.1% (50). The majority of pulmonary embolisms were associated with a significantly high level of inflammatory markers and cytokine storm.

The overall mortality of pulmonary embolism patients, if left untreated, is around 30%. Similar to this study, the mortality rate of PE-positive COVID-19 patients was 25% (16). There may be several complex and varied coagulation abnormalities in COVID-19 patients that produce a hypercoagulable state. The pathogenesis of hypercoagulability in COVID-19 patients may be caused by direct endothelial cell invasion by the SARS-CoV-2 virus, leading to complement-mediated endothelial injury, microvascular inflammation, endothelial exocytosis, and endotheliitis [18-21]. Stasis of blood flow may be seen in all critically ill hospitalized and immobilized patients. Several changes in circulating prothrombotic factors, including elevated factor VII and fibrinogen, circulating prothrombotic microparticles, and neutrophil extracellular traps (NETs), have been reported in severe COVID-19 infection [22-24]. The elevated levels of degradation products of cross-linked fibrin, such as D-Dimer, have been observed to correlate with COVID-19 illness severity [25-26]. The hypercoagulable state is termed thrombo-inflammation or COVID-19-associated coagulopathy (CAC) and may increase the risk of VTE [27-28].

In a meta-analysis of 71 studies to investigate the incidence of PE on CT-pulmonary angiogram in COVID-19 patients, 17.9% (95% CI: 12%-23.8%) were pulmonary embolisms in the emergency department, 23.9% (95% CI: 15.2%-32.7%) in general wards, and 48.6% (95% CI: 41%-56.1%) in intensive care units. The most commonly involved area of pulmonary vasculature was peripheral as compared to central; 65.3% were peripheral (95% CI: 60%-70.1%), as opposed to central main pulmonary arteries, which were 32.9% (95% CI: 26.7%-39%) [29]. Another qualitative and quantitative analysis of three studies comprising data from 437 patients did not show statistically and significantly higher mortality in critically ill COVID-19 patients and concurrent PE. However, overall mortality was higher [30]. 

In this study, the majority of pulmonary embolism patients were in intensive care units and had critical or severe COVID-19 pneumonia. One-fourth (25%, 16) of PE-positive COVID-19 patients died. There is a significant Pearson correlation between CTPA-PE and PE clot burden severity of Qanadli score in percentages (p <0.001), as well as between severity COVID-19 pneumonia and discharge (p <0.022) in Table 4. There was other multiorgan involvement in COVID-19 patients, including skeletal muscle injury, acute myocardial injury, acute kidney injury, acute liver injury, and acute pancreatitis, in descending order (Table 3). There was no significant Pearson correlation between the severity of COVID-19 cytokine storm, PE clot burden severity, Qanadli score in percentages, and severe COVID-19 pneumonia.

The limitation of this study is retrospective observational data analysis. Enrolled patients were in intensive care units, and the majority had a critical illness (selection bias). In addition, in our study, the majority of patients were male. 

## Conclusions

In this retrospective observational study, there is a significant correlation between pulmonary embolism and high clot burden Qanadli CTPA scores, as well as between the severity of COVID-19 pneumonia and mortality. However, there was no significant correlation between high clot burden Qanadli CTPA scores, cytokine storm, and the clinical severity of COVID-19 pneumonia. These findings suggest that critically ill COVID-19 pneumonia with pulmonary embolism may have a higher mortality rate and a poorer prognostic marker in this cohort of patients. However, a large-scale study is warranted to further support our findings.
